# A-Lipoic Acid Alleviates Folic Acid-Induced Renal Damage Through Inhibition of Ferroptosis

**DOI:** 10.3389/fphys.2021.680544

**Published:** 2021-09-17

**Authors:** Xue Li, Yu Zou, Yuan-Yuan Fu, Jia Xing, Kai-Yue Wang, Peng-Zhi Wan, Xiao-Yue Zhai

**Affiliations:** ^1^Department of Histology and Embryology, Basic Medical College, China Medical University, Shenyang, China; ^2^Department of Nephrology, Shengjing Hospital of China Medical University, Shenyang, China; ^3^Department of Nephrology, First Affiliated Hospital of China Medical University, Shenyang, China; ^4^Institute of Nephropathology, China Medical University, Shenyang, China

**Keywords:** A-lipoic acid, ferroptosis, p53, folic acid, renal damage

## Abstract

Folic acid (FA)-induced acute kidney injury (AKI) is characterized by the disturbance of redox homeostasis, resulting in massive tubular necrosis and inflammation. Α-lipoic acid (LA), as an antioxidant, has been reported to play an important role in renal protection, but the underlying mechanism remains poorly explored. The aim of this study is to investigate the protective effect of LA on FA-induced renal damage. Our findings showed that LA could ameliorate renal dysfunction and histopathologic damage induced by FA overdose injection. Moreover, FA injection induced severe inflammation, indicated by increased release of pro-inflammatory cytokines tumor necrosis factor (TNF)-α and IL-1β, as well as infiltration of macrophage, which can be alleviated by LA supplementation. In addition, LA not only reduced the cellular iron overload by upregulating the expressions of Ferritin and ferroportin (FPN), but also mitigated reactive oxygen species (ROS) accumulation and lipid peroxidation by increasing the levels of antioxidant glutathione (GSH) and glutathione peroxidase-4 (GPX4). More importantly, we found that LA supplementation could reduce the number of Terminal deoxynucleotidyl transferase dUTP nick end labeling (TUNEL)-positive tubular cells caused by FA, indicating that the tubular cell death mediated by ferroptosis may be inhibited. Further study demonstrated that LA supplementation could reverse the decreased expression of cystine/glutamate antiporter xCT (SLC7A11), which mediated GSH synthesis. What is more, mechanistic study indicated that p53 activation was involved in the inhibitory effect of SLC7A11 induced by FA administration, which could be suppressed by LA supplementation. Taken together, our findings indicated that LA played the protective effect on FA-induced renal damage mainly by inhibiting ferroptosis.

## Introduction

Acute Kidney Injury (AKI) is referred as a transient decline of renal function, which confers the severe clinical syndrome associated with high mortality (Bellomo et al., 2012). It has been reported that AKI affected more than 13.3 million patients with about 1.7 million deaths around the world each year, and the mortality was AKI stage-dependent (Brown et al., 2016). The in-hospital mortality was 5.1% for patients with stage 1 AKI, 13.7% for patients with stage 2 AKI, and 24.8% for patients with stage 3 AKI (Pinheiro et al., 2019). In addition, studies have shown that the 30- and 90-day mortality of the patients with AKI in ICU was much higher than that of the patients without AKI (Santos et al., 2019). Moreover, it has been estimated that nearly 2 million AKI patients each year cannot fully recover and have a high risk of progressing to chronic kidney disease (CKD; de Seigneux and Martin, 2017).

It is well-known that AKI can arise in various pathological conditions, such as drugs, toxicants, ischemia/reperfusion (I/R), obstruction, or sepsis, resulting in acute tubular necrosis (Ronco et al., 2019). Despite unpredictable in most cases, the occurrence of AKI is significantly higher in severe I/R injury after kidney transplant surgery, cisplatin for tumor chemotherapy, or contrast media for radiography and so on, especially for the susceptible individuals, including old age and patients with diabetes mellitus or CKD (Zhang et al., 2020). This highlighted the urgent need for novel therapeutic approaches that aims at preventing and/or reversing its sequelae (Zhao et al., 2018). Folic acid (FA)-induced AKI is one of the typical models simulating drug or toxicant-induced tubular injury, which is closely related to crystal formation in the tubule lumen and cellular oxidative stress, leading to massive inflammatory reaction and eventually tubular cell death (Martin-Sanchez et al., 2018).

Ferroptosis is a recently described form of programmed cell death caused by uncontrolled iron dependent lipid peroxidation that distinguishes it from traditional modalities of regulated cell death including apoptosis, necrosis, and autophagy (Li et al., 2020a). Moreover, ferroptosis is a critical pathophysiological event in FA-induced AKI, characterized by the imbalance of redox homeostasis and lethal lipid-based reactive oxygen species (ROS) generation (Martin-Sanchez et al., 2017). Normally, iron is indispensable for many physiological functions in organisms and can be stored in Ferritin, including Ferritin heavy chain (FTH) and Ferritin light chain (FTL; Hu et al., 2019). FTH, a ferroxidase enzyme, can convert Fe^2+^ to the ferric form (Fe^3+^) that sequesters free iron (Mumbauer et al., 2019). While under pathological conditions, oxygen reacts with excess labile iron and generates ROS that attacks lipid membrane, implicated in lipid peroxidation (Han et al., 2020). It has been demonstrated that proximal tubule cells expressing FTH could store iron and effectively limit free iron-mediated toxicity (Swaminathan, 2018). In contrast, the specific FTH gene knockout of the mouse proximal tubules could exacerbate renal damage in rhabdomyolysis or cisplatin-induced AKI (Zarjou et al., 2013). Moreover, iron overload could be alleviated by the only known iron exporter, ferroportin (FPN) that promotes iron transport out of cells and inhibits the production of ROS (Geng et al., 2018).

Additionally, the accumulation of lipid ROS is triggered when the endogenous antioxidant status is compromised. Glutathione (GSH) is an important antioxidant that protects against ferroptosis and it can be synthesized *via* cystine uptake mediated by cystine/glutamate antiporter xCT (Lim et al., 2019). Studies have shown that system xCT was mainly located in tubular cells, and its inhibition resulted in a rapid decline in GSH levels, which may accelerate AKI (Su et al., 2019). Inhibition of xCT by Class 1 ferroptosis inducers, such as erastin, may induce ferroptotic cell death mainly by depletion of cellular GSH content, and subsequently lead to inactivation of glutathione peroxidase-4 (GPX4; Yu et al., 2017). GPX4 is an essential lipid hydro-peroxide detoxifying enzyme, which can be directly inhibited by Class 2 ferroptosis inducers without affecting intracellular GSH. The inactivation of GPX4 in the proximal tubules could cause ferroptosis and further induce AKI (Yang et al., 2014). Apart from the features of ferroptosis mentioned above, TUNEL assay was often used to evaluate ferroptotic cells, labeling cells with DNA breakage (Cao et al., 2020).

P53, a tumor suppressor protein, is a critical regulator in various cell biological processes, mainly including cell cycle arrest and apoptosis, which is closely associated with AKI progression (Tang et al., 2019). P53 can also be induced in the oxidative stress response, sensitizing cells to ferroptosis *via* inhibiting system xCT (Zhang et al., 2018).

Lipoic acid (LA), a well-known antioxidant, has been reported to exert the protective effect on I/R-induced AKI by scavenging ROS (Bae et al., 2009). In addition, LA could alleviate oxidative damage to DNA through downregulation of p53 in Parkinson’s diseases (Chang et al., 2012). Moreover, LA was proved to chelate excess iron ions, thereby decreasing the risk of Alzheimer’s disease (Zhang et al., 2018). So far, we have known that the inhibition of oxidative stress is beneficial for alleviating FA-induced AKI, but the role of LA in this pathological process remains largely unknown. Therefore, the present study aimed to examine the effects of LA on FA-induced AKI and the underlying regulatory mechanisms. We observed that LA could prevent from FA-induced renal damage mainly by reducing ferroptosis.

## Materials and Methods

### Animals

The animal experiments were performed in accordance to the NIH Criteria for the Use of Laboratory Animals, and this study was approved by the ethics committee of the China Medical University Institutional Animal Care and Use Committee (protocol no. 2011037). C57BL/6J mice (male, 6–8week-old) were acquired from China Medical University (Liaoning, China). The animals were kept in housing facility at the temperature of 22°C with a 12-12h light–dark cycle. Mice were allocated into four groups randomly (*n*=6 per group): Control group, FA (250mg/kg at the concentration of 12.5mg/ml, dissolved in 300mM sodium bicarbonate) group, FA+LA-L (50mg/kg, dissolved in saline) group, and FA+LA-H (100mg/kg, dissolved in saline) group. Animals of the FA group were intraperitoneally injected with FA once. Animals of the treatment groups received oral medications of LA 24h before FA injection and continued afterward for 2days. On the second day after FA injection, samples of kidney specimens and blood were harvested for further examination. The mice were housed in the metabolic cage in the last 24h, allowing quantitative urine collection.

### Reagents and Antibodies

Folic acid and LA were provided by Dalian Meilun Biotechnology Co. (Dalian, China). Antibodies to F4/80, IL-1β, and p53 were obtained from CST (Danvers, United States). Antibodies to kidney injury molecule KIM-1, 4-HNE 4-hydroxynonenal, GPX4, xCT, Ferritin, FPN, and β-actin were purchased from Abcam (Cambridge, United States). Antibodies to TNF-α was obtained from Proteintech (Wuhan, China).

### Assays for Renal Function, ROS, GSH, and Iron

Blood Urea Nitrogen (BUN) and creatinine were measured following manufacturer’s instructions (Jian Cheng, Nanjing, China). ROS levels in kidney tissues were measured by DCFH-DA test kit. In brief, tissue homogenates were diluted 1:20 in cold buffer to obtain a concentration of 5mg tissue/ml. The reaction mixture containing the homogenate and DCFH-DA (5mmol/L) was incubated for 15min at room temperature. After 30min of further incubation, the conversion of DCFH-DA to the fluorescent product DCF was measured by a confocal laser scanning microscope with excitation at 488nm and an emission at 525nm to determine the concentration of ROS in the samples. Then ROS formation was quantified. Urinary sodium and potassium levels were determined using Quantichrom Assay Kits (BioAssay Sytems) and then fractional excretion of sodium (FENa) and fractional excretion of potassium (FEK) were calculated using the corresponding formulas (Ashour et al., 2016). Urinary osmolality were measured by the 5,004 Micro-Osmette (Precision Systems). Kidneys were homogenized to measure the levels of ROS (Solarbio, Beijing, China), GSH (Solarbio, Beijing, China), and iron content (Jian Cheng, Nanjing, China) according to manufacturer’s instructions.

### TUNEL Assay

To assess tubular cell death, TUNEL assay was used on kidney section embedded in paraffin according to the product’s protocols (Roche, Basel, Switzerland).

### Renal Histopathology

The paraffin-embedded kidneys were cut in 3-μm-thick slices for hematoxylin and eosin (H&E) and Periodic acid-Schiff (PAS) staining to evaluate the histopathologic injury. Furthermore, H&E-stained slices were used to assess acute tubular injury in a blinded way, including tubular epithelial vacuolization, dilation of tubular lumen, brush border loss, or cast formation. The criteria were based on semi-qualification as follows: (0) none; (1) <20%; (2) <20–50%; (3) <50–70%; and (4) >70% (Brooks et al., 2009).

### Prussian Iron Staining

Iron deposits in tubular cells were detected by Prussian iron staining (Abcam, Cambridge, MA, United States), as depicted in the book (American Registry of Pathology, Prophet, Edna B, 1992).

### Immunohistochemical Staining

Immunohistochemical (IHC) staining was performed in paraffin-embedded sections of 3-um thickness. The sections were deparaffinized, hydrated, antigen retrieved, and then probed with primary antibodies against KIM-1, IL-1β, TNF-α, F4/80, 4-HNE, GPX4, Ferritin, and p53 at the dilution of 1:200, followed by the incubation with the secondary antibodies for 60min on the second day. The sections were visualized with diaminobenzidine and counterstained with hematoxylin. Images were viewed using Nikon microscope (90i, Nikon, Tokyo, Japan).

### Western Blotting Analysis

Proteins from the whole kidney lysates were separated on a 10% SDS-polyacrylamide gel, transferred onto PVDF membrane, blocked in 5% BSA for 60min, and then primary antibodies against KIM-1, IL-1β, TNF-α, Ferritin, FPN, xCT, and p53 at the dilution of 1:1,000, as well as 4-HNE, GPX4, and β-actin at the dilution of 1:4,000 were probed at 4°C overnight. On the second day, peroxidase-conjugated secondary antibodies were probed for 60min at room temperature. The bands were visualized *via* chemiluminescence (ECL) system. Quantitative densitometry was used that the target proteins were analyzed using the Image software 6.0. The expression levels of proteins were normalized to that of β-actin.

### Real-Time PCR

Total RNA of kidney tissue was extracted by TRIzol method. The cDNA was synthesized through reverse transcription from RNA with PrimeScript RT reagent kit (Vazyme). Then PCR was performed using the SYBR-Green Master PCR Mix (Vazyme). The sequence of the primers used was as follows:

KIM-1 forward: 5'-ATCCCATCCCATACTCCTACAG-3' and reverse: 5'CGGAAGGCAACCACGCTTA;

p53 forward: 5'-TGCTCACCCTGGCTAAAGTT-3'and reverse: 5'-GTCCATGCAGTGAGGTGATG-3';

β-actin forward: 5'-GGCTGTATTCCCCTCCATCG-3' and reverse: 5'-CCAGTTGGTAATGCCATGT-3'.

The relative gene expression level was assessed using the 2^−ΔΔCt^ method.

### Statistical Analysis

The data were presented as means ± SDs. Statistical analysis was performed with the statistical software, SPSS version 21.0. Comparisons were analyzed using one-way ANOVA followed by Bonferroni test. Statistical significance of difference was defined as a value of *p*<0.05.

## Results

### LA Supplementation Ameliorated Histopathologic Damage and Renal Dysfunction in FA-Induced AKI

**Figure 1:**
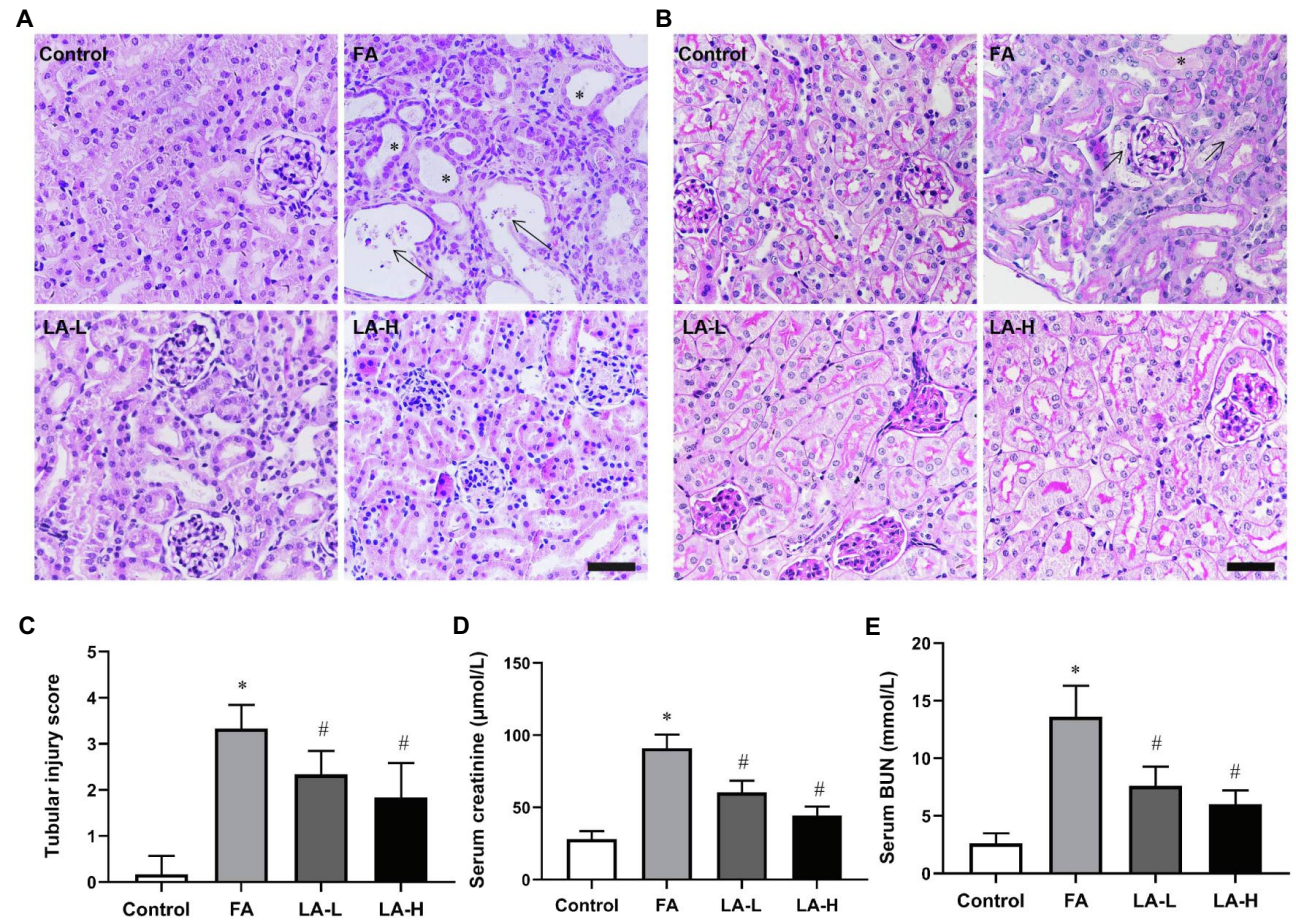
The protective effects of lipoic acid (LA) supplementation on folic acid (FA)-induced acute kidney injury (AKI). FA group mice are given an intraperitoneal injection of FA at 250mg/kg body weight once, without or with LA-L (50mg/kg body weight) or LA-H (100mg/kg body weight) supplementation. **(A)** Representative images of hematoxylin and eosin (H&E) staining, showing the histological changes in FA-induced AKI and the LA-treated groups. Bar=50μm. Asterisks for dilated tubule; arrows for necrotic tubular epithelial cells. **(B)** Renal damage is assessed by Periodic acid-Schiff (PAS) staining. Bar=50μm. Asterisks for tubular ectasia and arrows for tubules with cells in necrosis and cellular debris. **(C)** Renal tubular injury scores on the basis of H&E staining. The renal function is evaluated by **(D)** serum creatinine and **(E)** serum blood urea nitrogen (BUN) levels. For the FA group vs. the control group, *indicates *p*<0.05, and ^**^indicates *p*<0.01. For the LA-treated groups vs. the FA group, ^#^indicates *p*<0.05, and ^##^indicates *p*<0.01.

To determine whether LA supplementation could protect against FA-induced renal damage, histological analysis was evaluated with H&E and PAS staining. As observed in H&E staining, FA injection induced severe tubular damage, indicated by detachment of tubular cells, dilation of tubules, swelling of tubular cells, degeneration of epithelial cells, and interstitial inflammatory cells infiltration. In line with this tubular damage, the kidney in the FA group also showed aggravated pathological damage by PAS staining, characterized by necrotic tubular epithelial cells, tubular cast, and ablation of brush. While these tubular damage were alleviated by LA supplementation. Moreover, tubular injuries were assessed by quantification of HE-stained sections and revealed significantly increased tubulointerstitial injury scores in mice injected with FA injection, which were reduced by LA supplementation in a dose-dependent manner, but without statistical significance. In addition, renal function was measured to investigate the protective effect of LA on FA-induced AKI. Consistently, the FA injection induced dramatic increases in serum creatinine and BUN levels, indicating impaired renal function, while these functional parameters associated with AKI were reduced by LA supplementation, indicating the amelioration of renal function. The results above are summarized in Figure 1.

In addition, to fully determine the impact of LA on renal dysfunction induced by FA, the direct evaluation of renal function was determined with the indicators as displayed in Supplementary Table 1, FA injection markedly decreased urine volume, glomerular filtration rate (GFR), and filtration fraction compared with control mice, but these indexes could be reversed partially by LA supplementation, without obvious difference between the two dose groups. Moreover, the FENa, FEK, and urinary osmolarity were significantly reduced in FA-induced mice, which were remarkably reversed by LA supplementation.

**Figure 2:**
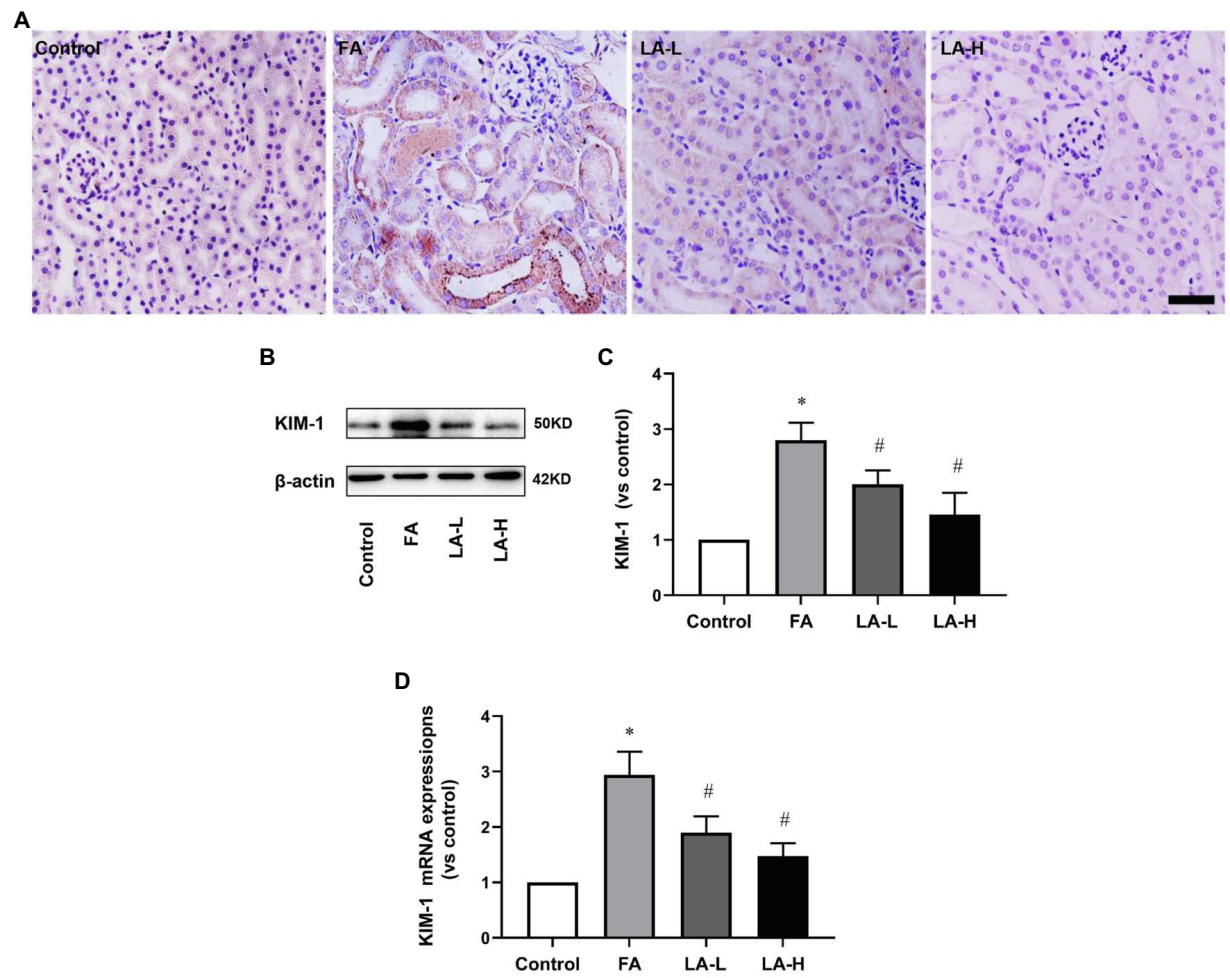
The effect of LA supplementation on acute tubular damage caused by FA injection. Mice are treated as described in Figure 1. **(A)** Representative images of immunohistochemical (IHC) staining for KIM-1. Bar=50μm. **(B)** Renal tubular damage marker, KIM-1 expression, as determined by western blotting. **(C)** Semi-quantitative assessments of KIM-1. **(D)** The expression of KIM-1 gene, as determined by relative mRNA expression. For the FA group vs. the control group, *indicates *p*<0.05, and **indicates *p*<0.01. For the LA-treated groups vs. the FA group, ^#^indicates *p*<0.05, and ^##^indicates *p*<0.01.

KIM-1, a biomarker of renal tubular damage, was examined by IHC staining. The results showed that the expression of KIM-1 in the tubular cells was upregulated after FA injection, while the effect was blocked by LA supplementation. Moreover, western blotting data confirmed that FA injection induced the increased expression of KIM-1, which was alleviated by LA supplementation in a dose-dependent manner, but no particular preference for the higher-dose group. Consistent with the protein levels, gene expression studies disclosed increased mRNA level of KIM-1 after FA injection, which was inhibited by LA supplementation. The results are summarized in Figure 2.

### LA Supplementation Alleviated Inflammation in FA-Induced AKI

**Figure 3:**
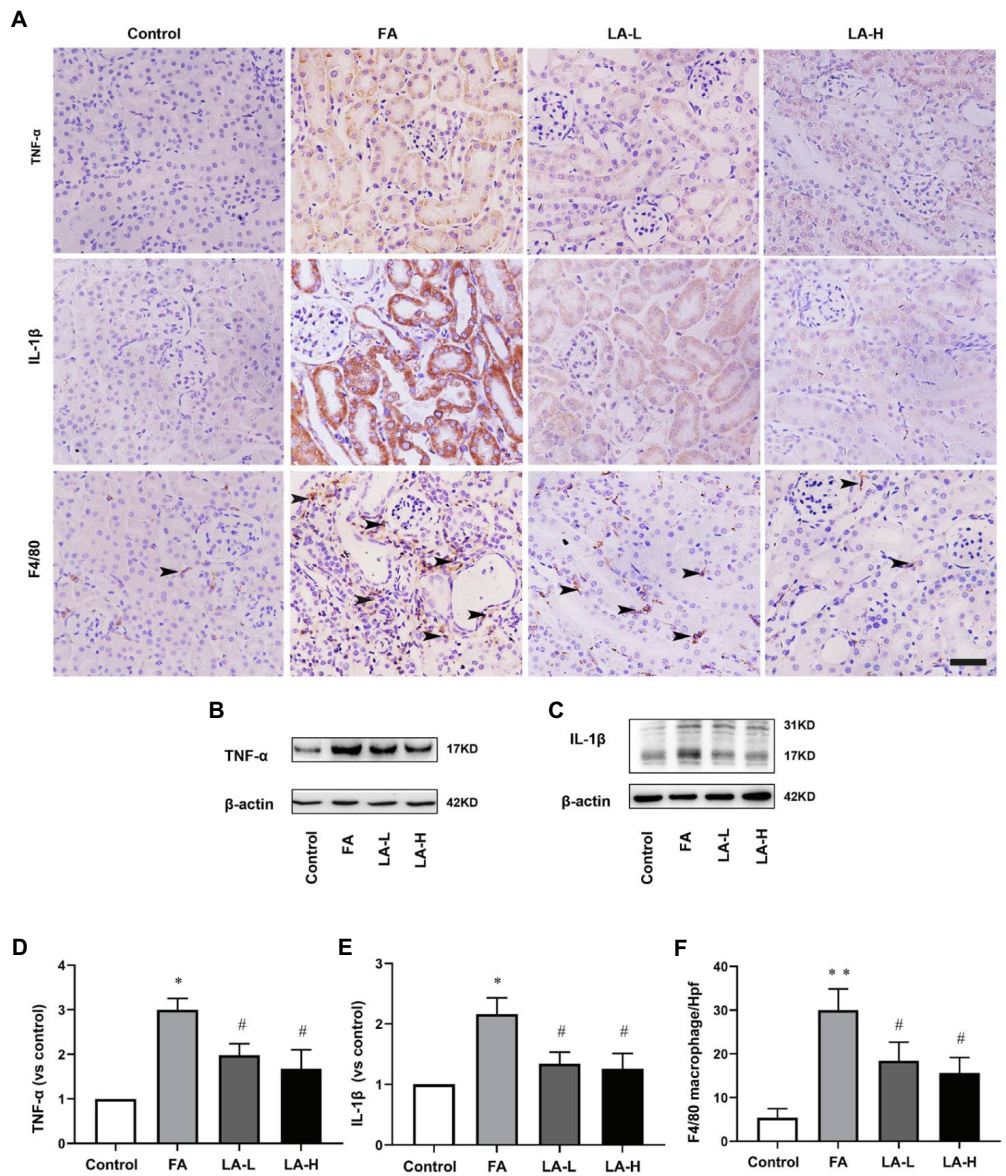
Lipoic acid supplementation reduces release of TNFα and IL-1β, as well as macrophage infiltration caused by FA injection. Mice are treated as described in Figure 1. **(A)** Representative images of IHC staining for TNF-α (brown), IL-1β (brown), and macrophages (brown). Bar=50μm. Black arrow heads for F4/80-positive interstitial macrophages. **(B)** The expression of inflammatory marker TNF-α, as determined by western blotting. **(C)** The expression of inflammatory marker IL-1β, as determined by western blotting. **(D)** Semi-quantitative assessments of TNF-α. **(E)** Semi-quantitative assessments of IL-1β. **(F)** The number of macrophages per high-power field (Hpf). For the FA group vs. the control group, *indicates *p*<0.05, and **indicates *p*<0.01. For the LA-treated groups vs. the FA group, ^#^indicates *p*<0.05, and ^##^indicates *p*<0.01.

Considering the key role of inflammation in FA-induced AKI, we evaluated the release of pro-inflammatory factors and the infiltration of inflammatory cells using IHC staining. The results showed that FA injection markedly enhanced the secretion of TNF-α and IL-1β in the tubular epithelium, together with the increased infiltration of macrophage in the interstitial space, whereas LA supplementation downregulated the levels of these molecules. To corroborate our IHC data, western blotting experiments for TNF-α and IL-1β showed that their expressions were significantly increased after FA injection, which were alleviated by LA supplementation but without significant difference between the two dose groups, shown in Figure 3.

### Effect of LA Supplementation on Iron Accumulation in FA-Induced AKI

Disorder of iron metabolism was the main cause of FA-induced AKI (Martin-Sanchez et al., 2017). To validate the protective effect of LA supplementation, iron deposits and distribution in the kidney was assessed. With iron staining where the deposits of iron were visualized, there were more iron-positive cells in the tubule of FA-injected kidney, while LA-treated kidney showed less iron-positive cells, compared to those normal cells in control group; But there was no statistical difference between the two LA-treated groups. Consistently, the content of iron in the kidney of LA-treated mice was less as compared to that in FA-injected mice.

**Figure 4:**
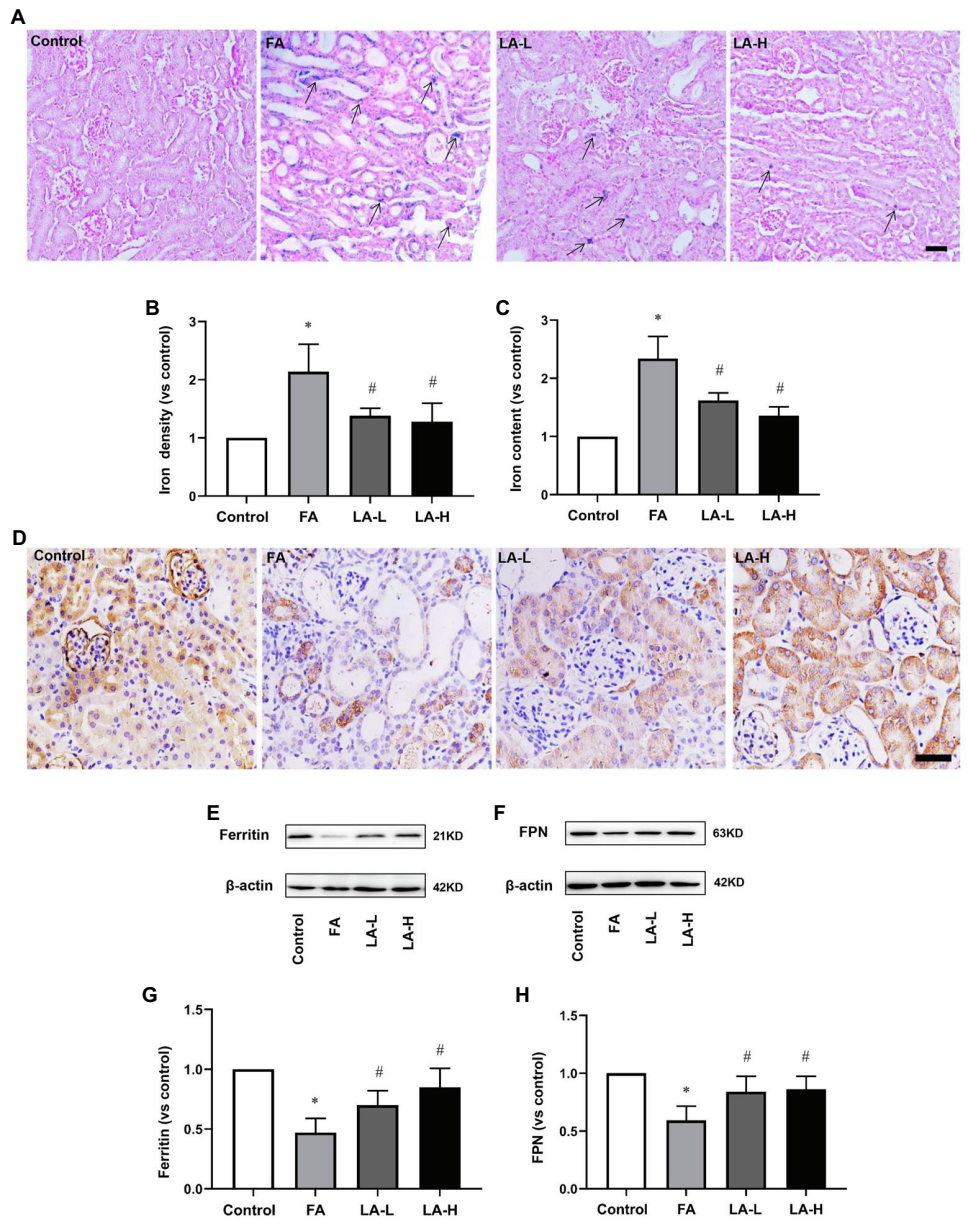
Lipoic acid supplementation alleviates iron accumulation caused by FA injection. Mice are treated as described in Figure 1. **(A)** Representative images of Perl’s staining. Arrows for iron accumulation. Bar=50μm. **(B)** Quantitative assessment of iron staining. **(C)** Iron content of kidney tissue. **(D)** Representative images of IHC staining for Ferritin (brown). **(E)** The expression of iron storage marker Ferritin, as determined by western blotting. **(F)** The expression of iron exporter marker ferroportin (FPN), as determined by western blotting. **(G)** Semi-quantitative assessments of Ferritin. **(H)** Semi-quantitative assessments of FPN. For the FA group vs. the control group, ^*^indicates *p*<0.05, and ^**^indicates *p*<0.01. For the LA-treated groups vs. the FA group, ^#^indicates *p*<0.05, and ^##^indicates *p*<0.01.

To explore the possible mechanism associated with the altered iron accumulation, the levels of Ferritin and FPN were analyzed. IHC staining revealed that Ferritin was diffusely distributed in the cytoplasm of tubular epithelial cells of the control mice, while Ferritin was significantly downregulated in FA-injected mice, which was reversed by LA supplementation. A similar result was observed in western blotting analysis that LA treated-mice in a dose-dependent trend increased Ferritin expression compared with FA-injected mice, but without obvious statistical significance between the two dose groups. Meanwhile, western blotting analysis showed that FA injection decreased the expression of FNP, which could be restored by LA supplementation. These results revealed that LA supplementation might enhance iron storage and promote iron turnover by upregulating Ferritin and FPN, therefore reducing iron accumulation, as shown in Figure 4.

### Effect of LA Supplementation on Anti-oxidative Stress in FA-Induced AKI

**Figure 5:**
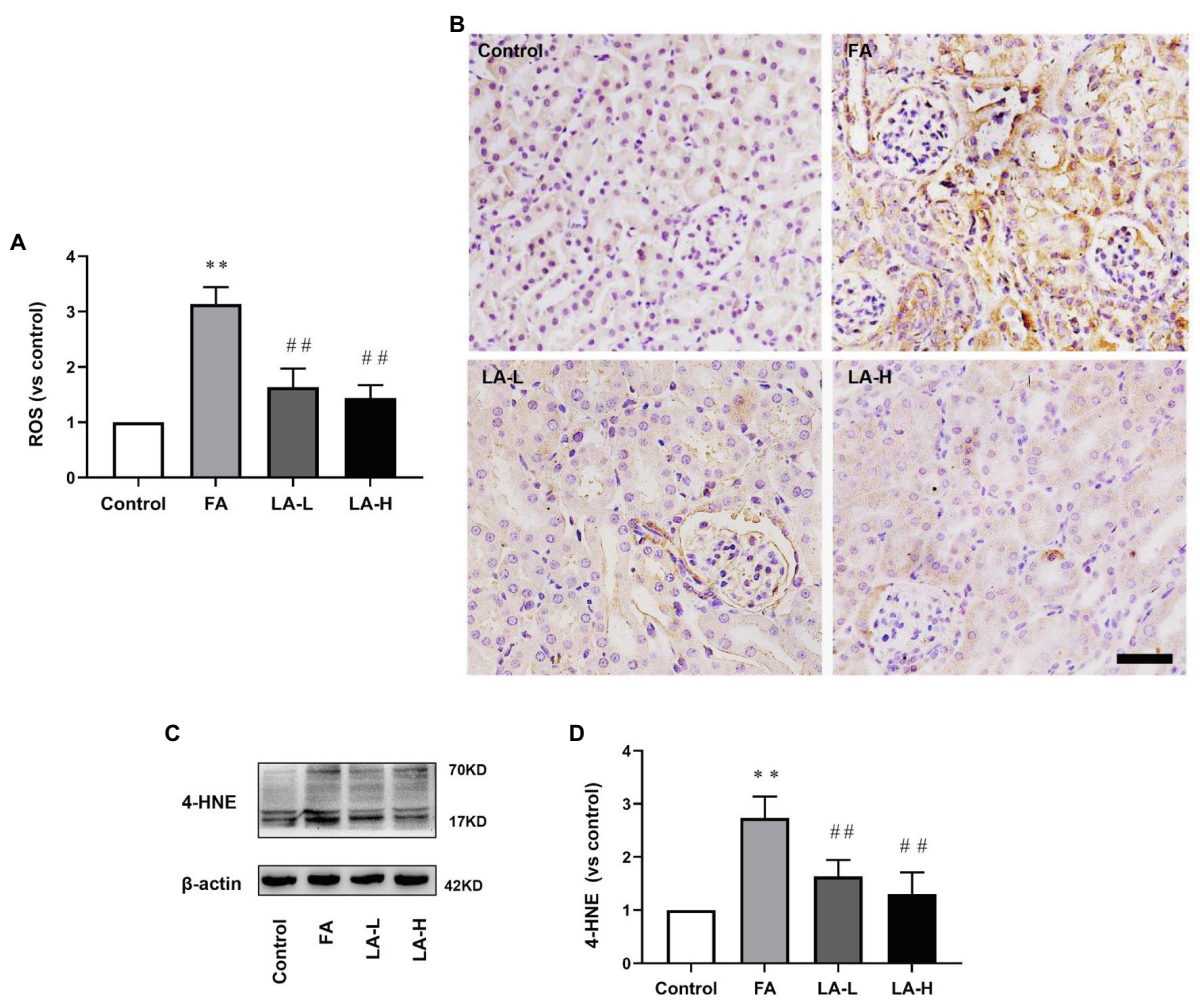
Lipoic acid supplementation decreases oxidative stress caused by FA injection. Mice are treated as described in Figure 1. **(A)** Reactive oxygen species (ROS) level in the kidney. **(B)** Representative images of IHC staining for 4-HNE (brown). Bar=50μm. **(C)** The expression of lipid peroxidation marker 4-HNE, as determined by western blotting. **(D)** Semi-quantitative assessments of 4-HNE. For the FA group vs. the control group, ^*^indicates *p*<0.05, and ^**^indicates *p*<0.01. For the LA-treated groups vs. the FA group, ^#^indicates *p*<0.05, and ^##^indicates *p*<0.01.

Oxidative stress has been demonstrated to be the key element to induce AKI (Li et al., 2020b), therefore, we further evaluated the levels of ROS in the kidney. The results showed that the production of ROS in LA-treated mice was lower than that in FA-injected mice. In addition, IHC staining demonstrated that the intensity of 4-HNE, reflecting the lipid perioxidation, was higher after FA injection, while LA supplementation significantly downregulated the intensity of 4-HNE. To address the issue, western blotting analysis was further performed to confirm that there were obvious decreased level of 4-HNE in LA-treated mice, compared with FA-injected ones, but no significant difference between the high- and low-dose groups was observed, as shown in Figure 5.

**Figure 6:**
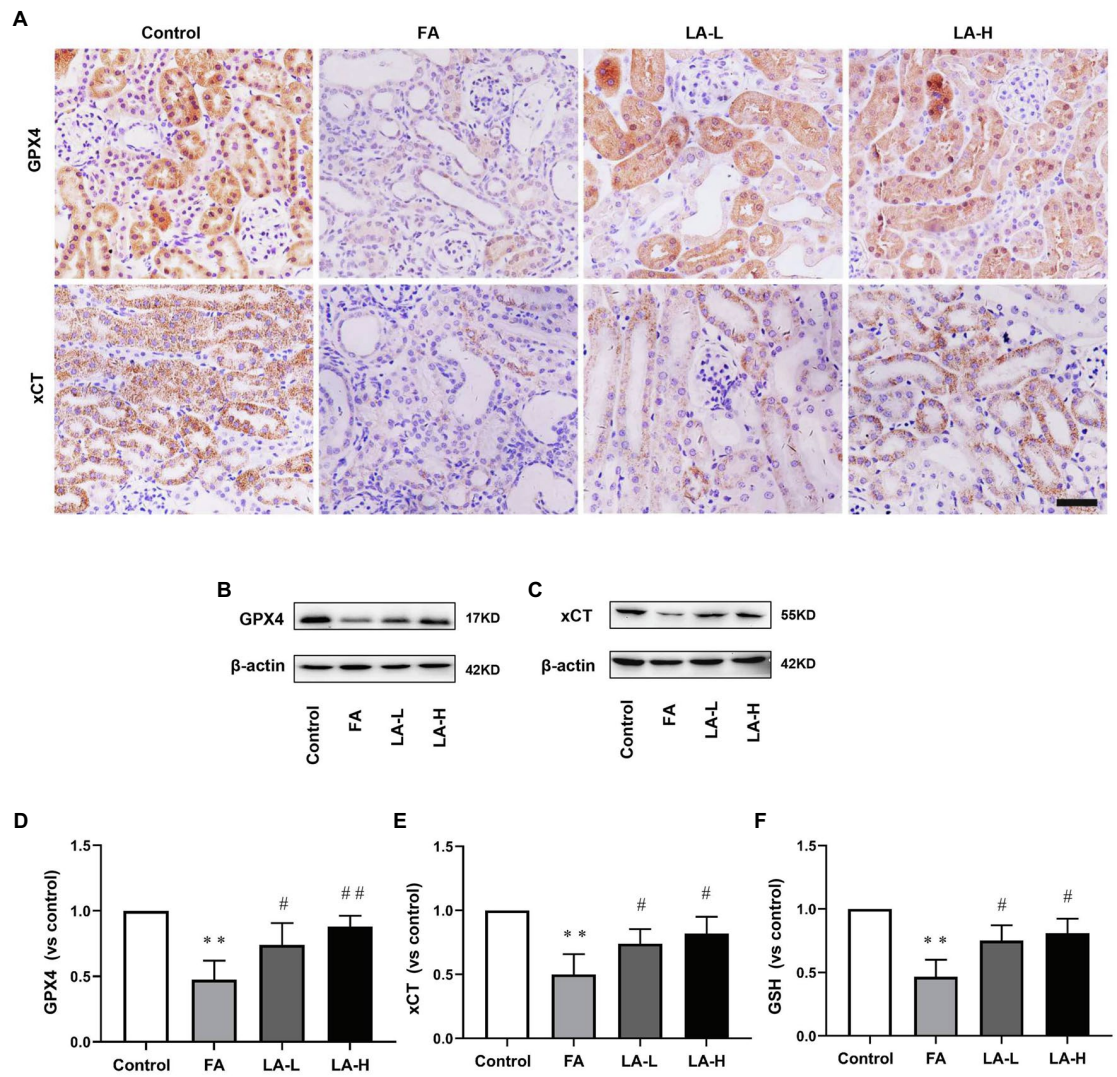
Lipoic acid supplementation increases FA-induced reduction in antioxidants. Mice are treated as described in Figure 1. **(A)** Representative images of IHC staining for GPX4 (brown) and xCT (brown). Bar=50μm. **(B)** The expression of anti-oxidative enzyme marker GPX4, as determined by western blotting. **(C)** The expression of cystine/glutamate transporter xCT, as determined by western blotting. **(D)** Semi-quantitative assessments of GPX4. **(E)** Semi-quantitative assessments of xCT. **(F)** The levels of antioxidant glutathione (GSH) content. For the FA group vs. the control group, ^*^indicates *p*<0.05, and ^**^indicates *p*<0.01. For the LA-treated groups vs. the FA group, ^#^indicates *p*<0.05, and ^##^indicates *p*<0.01.

We further examined the levels of GPX4 and xCT, two important antioxidants related to anti-oxidative stress. IHC staining analyses showed that GPX4 and xCT were mostly expressed in tubular epithelium, while FA injection reduced the levels of GPX4 and xCT, which can be reversed by LA supplementation. Similarly, western blotting analysis confirmed that the expression of GPX4 in LA treated-mice was upregulated in a dose-dependent manner, compared with downregulation in FA injected-mice, although there was no significant statistical difference between two dose groups. In addition, the xCT activity of mice treated with high-dose LA was enhanced compared with mice injected with FA, but there was no significant increase in activity compared with mice treated with low-dose LA. Furthermore, we evaluated the effect of LA on the content of GSH, the synthesis of which was mediated by xCT. The results showed a downregulated level of GSH in FA injection, and this effect was blocked by LA supplementation, consistent with the level of xCT, as shown in Figure 6.

Altogether, the results indicated that LA protected the kidney from FA-induced oxidative stress damage, which may be closely related to the maintenance of the antioxidant defenses system.

### Effect of LA Supplementation on Ferroptosis in FA-Induced AKI

**Figure 7:**
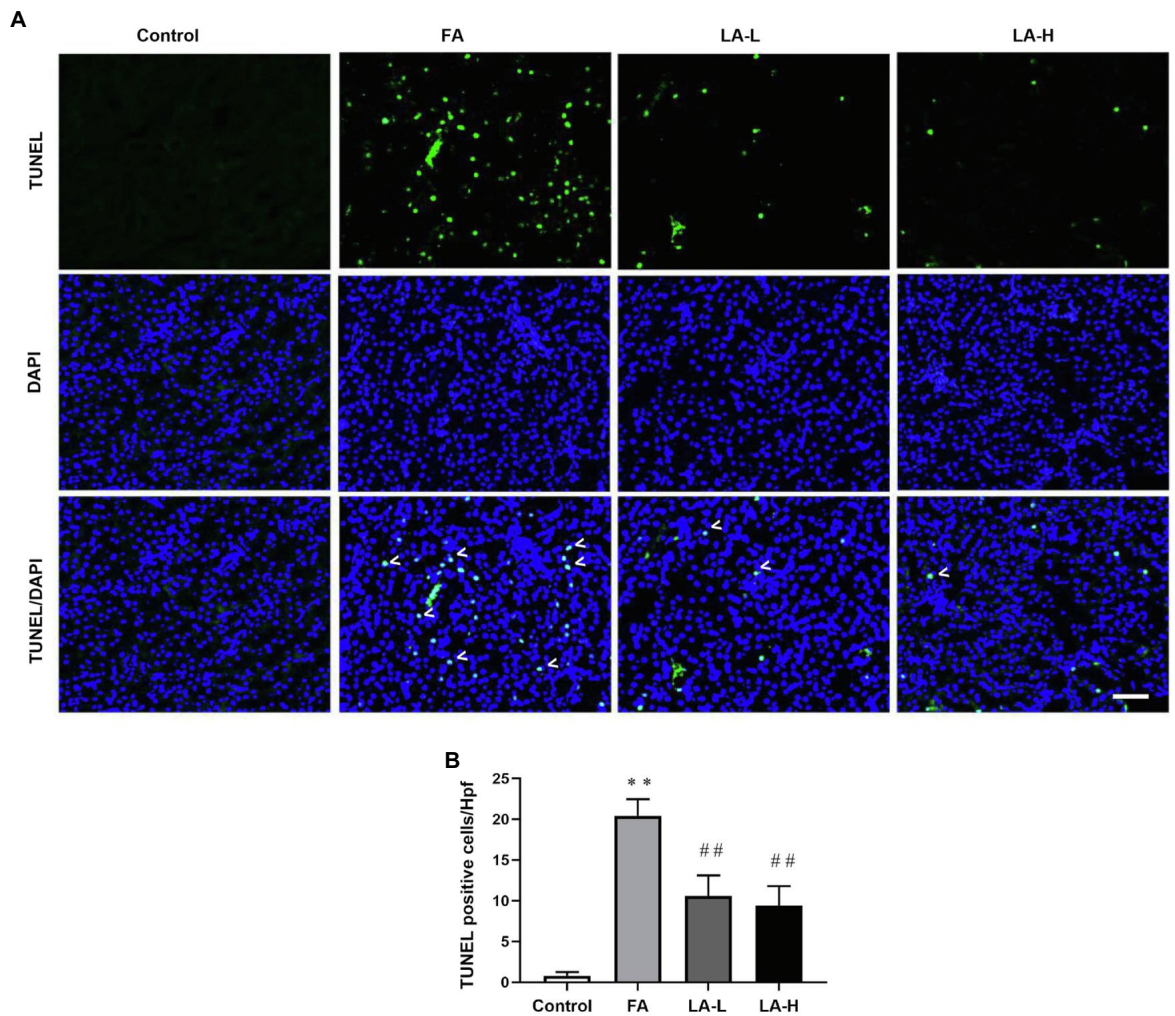
Lipoic acid supplementation decreases ferroptosis-mediated tubular cells death caused by FA injection. Mice are treated as described in Figure 1. **(A)** Representative images by TUNEL staining, showing the ferroptosis-mediated tubular cell death. White arrowheads for TUNEL-positive tubular cells (green) on kidney tissue sections; nuclei are labeled with DAPI (blue). Bar=50μm. **(B)** The number of TUNEL-positive nuclei per high-power field (Hpf). For the FA group vs. the control group, ^*^indicates *p*<0.05, and ^**^indicates *p*<0.01. For the LA-treated groups vs. the FA group, ^#^indicates *p*<0.05, and ^##^indicates *p*<0.01.

Iron-dependent lipid perioxidation could cause cell necrosis in tubular epithelium (Conrad et al., 2018). We further explored FA-induced tubular cell death using TUNEL staining. As depicted in Figure 7, we found that there were more TUNEL-positive tubular cells in the FA group, and the number of TUNEL-positive tubular cells in the low-dose and high-dose LA treatment groups was significantly reduced, while there was no particular preference for the higher-dose group. This indicated that LA could reduce ferroptosis-mediated tubular cell death without significant dose dependence.

### LA Supplementation Prevented FA-Induced xCT Reduction by Inhibiting p53 Activation

**Figure 8:**
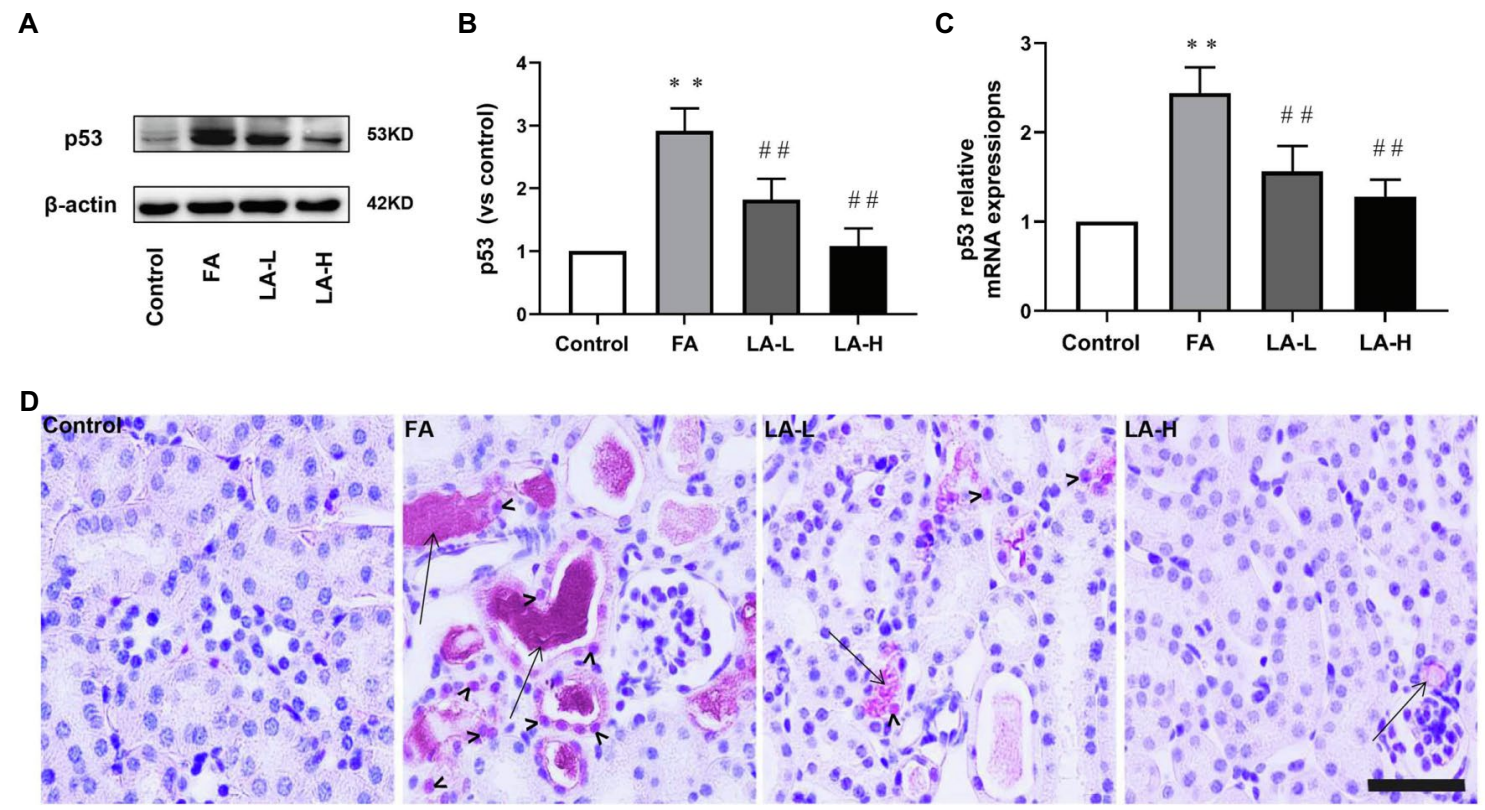
Lipoic acid supplementation inhibits the activation of p53 caused by FA injection. Mice are treated as described in Figure 1. **(A)** The expression for p53, as determined by western blotting. **(B)** Semi-quantitative assessments of p53. **(C)** The expression of p53 gene, as determined by relative mRNA expression. **(D)** Representative images of IHC staining for p53 activation. Bar=50μm. Black arrow heads for p53 nuclear staining; black arrows for folic acid crystals in tubular lumen. For the FA group vs. the control group, ^*^indicates *p*<0.05, and **indicates *p*<0.01. For the LA-treated groups vs. the FA group, ^#^indicates *p*<0.05, ^##^indicates *p*<0.01.

P53 served as a major factor of cellular ferroptosis that respond to various injuries, and we further examined whether LA can protect from ferroptosis that was associated with p53 activation in tubular epithelial cells. As shown in Figure 8, western blotting analyses indicated that FA injection elevated the expression of p53, which could be declined by LA supplementation in a dose-dependent manner. Meanwhile, the level of p53 mRNA displayed a similar response. Moreover, IHC staining showed that FA not only induced the expression of p53 but also promoted p53 abundance in nucleus. As compared to the FA group, LA supplementation significantly downregulated the trans-location of p53 into the nuclei. This implied that LA supplementation may increase xCT expression by inhibiting p53 activation.

## Discussion

A growing evidence has suggested that regulated necrosis could induce numerous inflammation and serves as a therapeutic target of AKI (Lin and Hsu, 2020). Ferroptosis, an oxidative stress-dependent programmed cell death, played an important role in FA-induced AKI (Martin-Sanchez et al., 2017; Li et al., 2020b). Moreover, FA-induced AKI was the main model of nephrotoxic AKI that was usually established by intraperitoneally injected FA at the dose of 250mg/kg (Diego et al., 2017), but the concentration was not demonstrated. In our study, FA 250mg/kg at the concentration of 12.5mg/ml was selected to induce AKI due to the higher concentration of FA (25 or 50mg/ml) could induce the death of mice.

Lipoic acid is a well-known antioxidative agent that can be used clinically to treat DM and neurodegenerative diseases, closely related to reducing the risk of occurrence of complication and mortality (Singh and Jialal, 2008; Molz and Schroder, 2017; Liakopoulos et al., 2019). What is more, LA has been used to protect against LPS or I/R induced AKI *via* anti-inflammation (Bae et al., 2009; Li et al., 2015; Zhang and McCullough, 2016). However, whether LA could exert the protective role in FA-induced AKI, its dosage and underlying mechanism remained unknown. In the present study, FA injection induced the production of massive inflammation. Meanwhile, FA administration could significantly increase the levels of FEK and FENa, probably due to the damage to proximal tubular cells and impaired Na^+^/K^+^-ATPase. In addition, a decrease was seen in urinary volume, GFR, and filtration fraction in the FA group, followed by an increase in the concentrations of BUN and Cr. While LA (50 or 100mg/kg) supplementation could significantly reduce histological damage and inflammation, and alters tubular water, sodium, and potassium handling, improving the renal function. It was in conjunction with the previous findings that treatment with LA (50mg/kg) could suppress UUO-induced tubular interstitial fibrosis in mice by ameliorating the epithelial mesenchymal transition (Hyun et al., 2016). In contrast, lower dose of LA (25mg/kg) was not found to ameliorate renal dysfunction in our study. Furthermore, the effect of LA (50mg/kg or 100mg/kg) on healthy animals was evaluated in the preliminary experiment, and no changes of renal morphology and function were found in the healthy mice after oral medications of LA (shown in Supplementary Figure 1).

Ferroptosis was the main tubular cell death that can significantly amplify inflammation. It is characterized by increased iron accumulation, leading to toxic ROS generation by the Fenton reaction (Lei et al., 2019). In our study, there was a large number of iron-positive staining tubular epithelial cells after FA over-injection. At the same time, the iron content in the kidney of FA-injected mice was statistically increment. Importantly, LA supplementation ameliorated renal injury accompanied with decreased iron accumulation. Furthermore, we demonstrated that the levels of Ferritin (a protein complex involved in intracellular iron storage) in the kidney were decreased after FA injection, while LA supplementation reversed the decreased levels. This indicated that the protective effects of LA on FA-induced AKI may increase iron storage by upregulation of Ferritin. Our study was in line with the previous study that upregulation of FTH could alleviate cisplatin induced HK2 cell injury through anti-ferroptosis (Hu et al., 2020). In addition, it has been reported that Ferritin degradation caused by increased autophagy can promote ferroptosis in fibroblasts and cancer cells (Hou et al., 2016). In addition, excessive iron could be exported through the iron efflux pump, FPN, thereby reducing intracellular iron overload (Geng et al., 2018). Accumulating evidences suggested the critical role of FPN in preventing neurodegenative disease caused by ferroptosis (Raha et al., 2013). Down this line, we observed that FA administration induced a transient decrease in FPN expression, while LA supplementation significantly upregulated FPN levels, thereby accelerating mobilization of excessive iron out of tubular cells and reducing intracellular iron overload. In this regard, LA may exert the effect of anti-ferroptosis through chelating intracellular excess iron and mediating iron turnover in tubule of the mouse kidney.

Of note, LA has been reported to act as a direct free radical scavenger that alleviated oxidative stress (Aruoma, 1998). In support of this, we demonstrated that LA supplementation could scavenge ROS and mitigate lipid peroxidation, which further reduced number of ferroptotic tubular cells. Furthermore, we found that LA could elevate the level of GSH, which was consistent with the research that macrophage migration inhibitory factor (MIF) increased intracellular GSH content which protected against oxidative damage of tubular epithelial cell *in vivo* and *in vitro* (Unruh et al., 2019). In addition, another study showed that deferoxamine could alleviate ferroptosis to promote recovery of traumatic spinal cord injury through upregulation of GSH (Yao et al., 2019). On the other hand, several investigators found that treatment with erastin could inhibit system xCT, thereby reducing extracellular cystine import for GSH synthesis and increasing the sensitivity to ferroptosis (Wang et al., 2020). Consistently, we found that the expression of xCT in the kidney was significantly downregulated induced by FA injection, which was closely related to the decrease in GSH levels. Moreover, inhibition of system xCT could lower the activity of GPX4, the vital antioxidant enzyme which allowed for blocking ferroptosis through reducing lipid hydroperoxides (Liu et al., 2017). It has been reported that GPX4 knockout mice could increase the sensitivity of the kidney to ferroptosis caused by IRI (Linkermann et al., 2014). Recovery of the expression of GPX4 has been demonstrated to alleviate ferroptosis in cardiomyocytes and neurons caused by I/R (Seibt et al., 2019; Stamenkovic et al., 2019). Correspondingly, our study found that ferroptosis induced by FA injection was accompanied by downregulation of system xCT, leading to the depletion of GSH, which in turn decreased the expression of GPX4.

Several studies indicated that p53 activation was associated with the inhibition of system xCT (Jiang et al., 2015). It was generally considered that p53 activity exerted anti-tumor effect by regulating apoptosis or cell-cycle arrest (Chen, 2016). In addition, the document has implicated that p53 expression was induced in response to cellular insults, which could accelerate neuron cell death (Morrison and Kinoshita, 2000). Emerging evidence validated that p53 activation played a critical role in mediating ferroptosis (Wang et al., 2016). Ling et al. discovered that inhibition of p53 could defend against liver fibrosis *via* reducing ferroptosis (Krstic et al., 2018). What is more, p53 activation in renal tubular epithelial cells was closely related to the deterioration of AKI (Zhang et al., 2014). As mentioned above, we explored the potential mechanisms of FA-induced system xCT suppression and found higher p53 activation in tubular cells induced by FA administration, while LA supplementation blocked the activity of p53 and abrogated the pro-ferroptosis effects. Therefore, p53-mediated ferroptosis in tubular epithelial cell may be a therapeutic target for alleviating AKI.

Taken together, our study demonstrated that ferroptosis played an important role in AKI induced by FA overdose injection in mice, which could be alleviated by LA supplementation, indicating that LA may be used as a potential therapeutic approach to ameliorate AKI caused by drugs or toxicants in the clinic. In our study, LA could be used as an anti-ferroptosis agent to reduce iron overload caused by FA injection through upregulation of Ferritin and FPN. Besides, LA supplementation could restore of the expression of system xCT, thereby promoting the synthesis of GSH and subsequently enhancing the activity of GPX4. Finally, we identified that inactivation of p53 was the key mechanism involved in restoring the expression of system xCT, thus suggesting the beneficial effects of LA on anti-ferroptosis in FA-induced AKI.

## Data Availability Statement

The raw data supporting the conclusions of this article will be made available by the authors, without undue reservation.

## Ethics Statement

The animal study was reviewed and approved by China Medical University.

## Author Contributions

XL wrote the manuscript. X-YZ conceived and designed the experiments. YZ analyzed the data. Y-YF collected and provided the sample for this study. JX revised this manuscript. K-YW did the statistics. All authors contributed to the article and approved the submitted version.

## Funding

The work was financially supported by the National Natural Science Foundation of China (contract no. 31971115).

## Conflict of Interest

The authors declare that the research was conducted in the absence of any commercial or financial relationships that could be construed as a potential conflict of interest.

## Publisher’s Note

All claims expressed in this article are solely those of the authors and do not necessarily represent those of their affiliated organizations, or those of the publisher, the editors and the reviewers. Any product that may be evaluated in this article, or claim that may be made by its manufacturer, is not guaranteed or endorsed by the publisher.

## Supplementary Material

Supplementary Table 1 Changes in renal function parameters due to FA overdose injection in mice and effect of LA supplementation. Data are collected after housing the mice in metabolic cages for the last 24h. Urinary volume are obtained in individual metabolic cage. GFR mean glomerular filtration rate; Flitration fraction=GFR/plasma flow; and FENa mean fractional excretion of sodium and FEK mean fractional excretion of potassium. Data are presented as mean±SE (*N*=5). For the FA group vs. the control group, ^*^indicates *p*<0.05, and ^**^indicates *p*<0.01. For the LA-treated groups vs. the FA group, #indicates p<0.05, and ##indicates p<0.01.

Supplementary Figure 1 No changes of renal morphology and function in the healthy mice after oral medications of LA (50 or 100mg/kg). The healthy mice are given an oral administration of LA at 50mg/kg (LA-50) or 100mg/kg (LA-100). **(A)** Representative images of H&E staining, showing histological changes of healthy mice without or with LA 50mg/kg (LA-50) or LA 100mg/kg (LA-100) oral administration. **(B)** Representative images of PAS staining, showing histological changes of healthy mice without or with LA 50mg/kg (LA-50) or LA 100mg/kg (LA-100) oral administration. The renal function is evaluated by **(C)** serum creatinine and **(D)** serum BUN levels.

Supplementary Figure 2 The original data of the western blotting.

## References

[ref1] Aruoma O. I. (1998). Free radicals, oxidative stress, and antioxidants in human health and disease. J. Am. Oil Chem. Soc. 75, 199–212. doi: 10.1007/s11746-998-0032-9, PMID: 32287334 PMC7101596

[ref2] Ashour R. H. Saad M. A. Sobh M. A. Al-Husseiny F. Abouelkheir M. Awad A.. (2016). Comparative study of allogenic and xenogeneic mesenchymal stem cells on cisplatin-induced acute kidney injury in Sprague-Dawley rats. Stem Cell Res. Ther. 7:126. doi: 10.1186/s13287-016-0386-0, PMID: 27585525 PMC5009659

[ref3] Bae E. H. Lee J. Ma S. K. Kim I. J. Frokiaer J. Nielsen S.. (2009). Alpha-Lipoic acid prevents cisplatin-induced acute kidney injury in rats. Nephrol. Dial. Transplant. 24, 2692–2700. doi: 10.1093/ndt/gfp176, PMID: 19376830

[ref4] Bellomo R. Kellum J. A. Ronco C. (2012). Acute kidney injury. Lancet 380, 756–766. doi: 10.1016/S0140-6736(11)61454-2, PMID: 22617274

[ref001] Brooks C. Wei Q. Cho S. G. Dong Z.. (2009). Regulation of mitochondrial dynamics in acute kidney injury in cell culture and rodent models. J. Clin. Invest. 119, 1275–1285. 19349686 10.1172/JCI37829 PMC2673870

[ref5] Brown J. R. Hisey W. M. Marshall E. J. Likosky D. S. Nichols E. L. Everett A. D.. (2016). Acute kidney injury severity and long-term readmission and mortality After cardiac surgery. Ann. Thorac. Surg. 102, 1482–1489. doi: 10.1016/j.athoracsur.2016.04.020, PMID: 27319985 PMC5077656

[ref6] Cao J. Chen X. Jiang L. Lu B. Yuan M. Zhu D.. (2020). DJ-1 suppresses ferroptosis through preserving the activity of S-adenosyl homocysteine hydrolase. Nat. Commun. 11:1251. doi: 10.1038/s41467-020-15109-y, PMID: 32144268 PMC7060199

[ref7] Chang J. R. Ghafouri M. Mukerjee R. Bagashev A. Chabrashvili T. Sawaya B. E. (2012). Role of p53 in neurodegenerative diseases. Neurodegener. Dis. 9, 68–80. doi: 10.1159/000329999, PMID: 22042001 PMC3304514

[ref8] Chen J. (2016). The cell-cycle arrest and apoptotic functions of p53 in tumor initiation and progression. Cold Spring Harb. Perspect. Med. 6:a026104. doi: 10.1101/cshperspect.a026104, PMID: 26931810 PMC4772082

[ref002] Cho H. S. Kim J. H. Jang H. N. Lee T. W. Jung M. H. Kim T. H.. (2017). Alpha-lipoic acid ameliorates the epithelial mesenchymal transition induced by unilateral ureteral obstruction in mice. Sci. Rep. 7:46065 28378840 10.1038/srep46065 PMC5380949

[ref9] Conrad M. Kagan V. E. Bayir H. Pagnussat G. C. Head B. Traber M. G.. (2018). Regulation of lipid peroxidation and ferroptosis in diverse species. Genes Dev. 32, 602–619. doi: 10.1101/gad.314674.118, PMID: 29802123 PMC6004068

[ref10] de Seigneux S. Martin P. Y. (2017). Preventing the progression of AKI to CKD: the role of mitochondria. J. Am. Soc. Nephrol. 28, 1327–1329. doi: 10.1681/ASN.2017020146, PMID: 28336720 PMC5407741

[ref11] Geng N. Shi B. J. Li S. L. Zhong Z. Y. Li Y. C. Xua W. L.. (2018). Knockdown of ferroportin accelerates erastin-induced ferroptosis in neuroblastoma cells. Eur. Rev. Med. Pharmacol. Sci. 22, 3826–3836. doi: 10.26355/eurrev_201806_15267, PMID: 29949159

[ref12] Han C. Liu Y. Dai R. Ismail N. Su W. Li B. (2020). Ferroptosis and its potential role in human diseases. Front. Pharmacol. 11:239. doi: 10.3389/fphar.2020.00239, PMID: 32256352 PMC7090218

[ref13] Hou W. Xie Y. Song X. Sun X. Lotze M. T. Zeh H. J. 3rd. (2016). Autophagy promotes ferroptosis by degradation of ferritin. Autophagy 12, 1425–1428. doi: 10.1080/15548627.2016.1187366, PMID: 27245739 PMC4968231

[ref14] Hu Z. Zhang H. Yang S. K. Wu X. He D. Cao K.. (2019). Emerging role of Ferroptosis in acute kidney injury. Oxidative Med. Cell. Longev. 2019:8010614. doi: 10.1155/2019/8010614, PMID: 31781351 PMC6875218

[ref15] Hu Z. Zhang H. Yi B. Yang S. Liu J. Hu J.. (2020). VDR activation attenuate cisplatin induced AKI by inhibiting ferroptosis. Cell Death Dis. 11:73. doi: 10.1038/s41419-020-2256-z, PMID: 31996668 PMC6989512

[ref16] Jiang L. Kon N. Li T. Wang S. J. Su T. Hibshoosh H.. (2015). Ferroptosis as a p53-mediated activity during tumour suppression. Nature 520, 57–62. doi: 10.1038/nature14344, PMID: 25799988 PMC4455927

[ref17] Krstic J. Galhuber M. Schulz T. J. Schupp M. Prokesch A. (2018). p53 as a dichotomous regulator of liver disease: the dose makes the medicine. Int. J. Mol. Sci. 19:921. doi: 10.3390/ijms19030921, PMID: 29558460 PMC5877782

[ref18] Lei P. Bai T. Sun Y. (2019). Mechanisms of Ferroptosis and relations With regulated cell death: A review. Front. Physiol. 10:139. doi: 10.3389/fphys.2019.00139, PMID: 30863316 PMC6399426

[ref19] Li G. Fu J. Zhao Y. Ji K. Luan T. Zang B. (2015). Alpha-lipoic acid exerts anti-inflammatory effects on lipopolysaccharide-stimulated rat mesangial cells via inhibition of nuclear factor kappa B (NF-kappaB) signaling pathway. Inflammation 38, 510–519. doi: 10.1007/s10753-014-9957-3, PMID: 24962643

[ref20] Li Y. Xia W. Wu M. Yin J. Wang Q. Li S.. (2020b). Activation of GSDMD contributes to acute kidney injury induced by cisplatin. Am. J. Physiol. Ren. Physiol. 318, F96–F106. doi: 10.1152/ajprenal.00351.2019, PMID: 31682173

[ref21] Li X. Zou Y. Xing J. Fu Y. Y. Wang K. Y. Wan P. Z.. (2020a). Pretreatment with Roxadustat (FG-4592) attenuates folic acid-induced kidney injury through Antiferroptosis via Akt/GSK-3beta/Nrf2 pathway. Oxidative Med. Cell. Longev. 2020:6286984. doi: 10.1155/2020/6286984, PMID: 32051732 PMC6995323

[ref22] Liakopoulos V. Roumeliotis S. Bozikas A. Eleftheriadis T. Dounousi E. (2019). Antioxidant supplementation in renal replacement therapy patients: is there evidence? Oxidative Med. Cell. Longev. 2019:9109473. doi: 10.1155/2019/9109473, PMID: 30774749 PMC6350615

[ref23] Lim J. K. M. Delaidelli A. Minaker S. W. Zhang H. F. Colovic M. Yang H.. (2019). Cystine/glutamate antiporter xCT (SLC7A11) facilitates oncogenic RAS transformation by preserving intracellular redox balance. Proc. Natl. Acad. Sci. U. S. A. 116, 9433–9442. doi: 10.1073/pnas.1821323116, PMID: 31000598 PMC6511045

[ref24] Lin T. Y. Hsu Y. H. (2020). IL-20 in acute kidney injury: role in pathogenesis and potential as a therapeutic target. Int. J. Mol. Sci. 21:1009. doi: 10.3390/ijms21249751, PMID: 32028746 PMC7037658

[ref25] Linkermann A. Skouta R. Himmerkus N. Mulay S. R. Dewitz C. De Zen F.. (2014). Synchronized renal tubular cell death involves ferroptosis. Proc. Natl. Acad. Sci. U. S. A. 111, 16836–16841. doi: 10.1073/pnas.1415518111, PMID: 25385600 PMC4250130

[ref26] Liu D. S. Duong C. P. Haupt S. Montgomery K. G. House C. M. Azar W. J.. (2017). Inhibiting the system xC(−)/glutathione axis selectively targets cancers with mutant-p53 accumulation. Nat. Commun. 8:14844. doi: 10.1038/s41467-017-02320-7, PMID: 28348409 PMC5379068

[ref27] Martin-Sanchez D. Fontecha-Barriuso M. Carrasco S. Sanchez-Nino M. D. Massenhausen A. V. Linkermann A.. (2018). TWEAK and RIPK1 mediate a second wave of cell death during AKI. Proc. Natl. Acad. Sci. U. S. A. 115, 4182–4187. doi: 10.1073/pnas.1716578115, PMID: 29588419 PMC5910825

[ref28] Martin-Sanchez D. Ruiz-Andres O. Poveda J. Carrasco S. Cannata-Ortiz P. Sanchez-Nino M. D.. (2017). Ferroptosis, but not Necroptosis, is important in nephrotoxic folic acid-induced AKI. J. Am. Soc. Nephrol. 28, 218–229. doi: 10.1681/ASN.2015121376, PMID: 27352622 PMC5198282

[ref29] Molz P. Schroder N. (2017). Potential therapeutic effects of Lipoic acid on memory deficits related to aging and Neurodegeneration. Front. Pharmacol. 8:849. doi: 10.3389/fphar.2017.00849, PMID: 29311912 PMC5732919

[ref30] Morrison R. S. Kinoshita Y. (2000). The role of p53 in neuronal cell death. Cell Death Differ. 7, 868–879. doi: 10.1038/sj.cdd.4400741, PMID: 11279532

[ref31] Mumbauer S. Pascual J. Kolotuev I. Hamaratoglu F. (2019). Ferritin heavy chain protects the developing wing from reactive oxygen species and ferroptosis. PLoS Genet. 15:e1008396. doi: 10.1371/journal.pgen.1008396, PMID: 31568497 PMC6786644

[ref32] Pinheiro K. H. E. Azedo F. A. Areco K. C. N. Laranja S. M. R. (2019). Risk factors and mortality in patients with sepsis, septic and non septic acute kidney injury in ICU. J. Bras. Nefrol. 41, 462–471. doi: 10.1590/2175-8239-JBN-2018-0240, PMID: 31528980 PMC6979581

[ref33] Raha A. A. Vaishnav R. A. Friedland R. P. Bomford A. Raha-Chowdhury R. (2013). The systemic iron-regulatory proteins hepcidin and ferroportin are reduced in the brain in Alzheimer's disease. Acta Neuropathol. Commun. 1:55. doi: 10.1186/2051-5960-1-55, PMID: 24252754 PMC3893417

[ref34] Ronco C. Bellomo R. Kellum J. A. (2019). Acute kidney injury. Lancet 394, 1949–1964. doi: 10.1016/S0140-6736(19)32563-2, PMID: 31777389

[ref35] Santos R. P. D. Carvalho A. R. S. Peres L. A. B. Ronco C. Macedo E. (2019). An epidemiologic overview of acute kidney injury in intensive care units. Rev. Assoc. Med. Bras. 65, 1094–1101. doi: 10.1590/1806-9282.65.8.1094, PMID: 31531608

[ref36] Seibt T. M. Proneth B. Conrad M. (2019). Role of GPX4 in ferroptosis and its pharmacological implication. Free Radic. Biol. Med. 133, 144–152. doi: 10.1016/j.freeradbiomed.2018.09.014, PMID: 30219704

[ref37] Singh U. Jialal I. (2008). Alpha-lipoic acid supplementation and diabetes. Nutr. Rev. 66, 646–657. doi: 10.1111/j.1753-4887.2008.00118.x, PMID: 19019027 PMC2657658

[ref38] Stamenkovic A. Pierce G. N. Ravandi A. (2019). Phospholipid oxidation products in ferroptotic myocardial cell death. Am. J. Physiol. Heart Circ. Physiol. 317, H156–H163. doi: 10.1152/ajpheart.00076.2019, PMID: 31050558

[ref39] Su L. Jiang X. Yang C. Zhang J. Chen B. Li Y.. (2019). Pannexin 1 mediates ferroptosis that contributes to renal ischemia/reperfusion injury. J. Biol. Chem. 294, 19395–19404. doi: 10.1074/jbc.RA119.010949, PMID: 31694915 PMC6916502

[ref40] Swaminathan S. (2018). Iron homeostasis pathways as therapeutic targets in acute kidney injury. Nephron 140, 156–159. doi: 10.1159/000490808, PMID: 29982259 PMC6165684

[ref41] Tang C. Ma Z. Zhu J. Liu Z. Liu Y. Liu Y.. (2019). P53 in kidney injury and repair: mechanism and therapeutic potentials. Pharmacol. Ther. 195, 5–12. doi: 10.1016/j.pharmthera.2018.10.013, PMID: 30347214

[ref42] Unruh M. Wagner B. Hallows K. R. (2019). MIF matters: the macrophage migration inhibitory factor and kidney injury. Am. J. Kidney Dis. 73, 429–431. doi: 10.1053/j.ajkd.2018.07.003, PMID: 30241958 PMC6389413

[ref43] Wang S. J. Li D. Ou Y. Jiang L. Chen Y. Zhao Y.. (2016). Acetylation is crucial for p53-mediated Ferroptosis and tumor suppression. Cell Rep. 17, 366–373. doi: 10.1016/j.celrep.2016.09.022, PMID: 27705786 PMC5227654

[ref44] Wang L. Liu Y. Du T. Yang H. Lei L. Guo M.. (2020). ATF3 promotes erastin-induced ferroptosis by suppressing system xc(.). Cell Death Differ. 27, 662–675. doi: 10.1038/s41418-019-0380-z, PMID: 31273299 PMC7206049

[ref45] Yang W. S. SriRamaratnam R. Welsch M. E. Shimada K. Skouta R. Viswanathan V. S.. (2014). Regulation of ferroptotic cancer cell death by GPX4. Cell 156, 317–331. doi: 10.1016/j.cell.2013.12.010, PMID: 24439385 PMC4076414

[ref46] Yao X. Zhang Y. Hao J. Duan H. Q. Zhao C. X. Sun C.. (2019). Deferoxamine promotes recovery of traumatic spinal cord injury by inhibiting ferroptosis. Neural Regen. Res. 14, 532–541. doi: 10.4103/1673-5374.245480, PMID: 30539824 PMC6334606

[ref47] Yu H. Guo P. Xie X. Wang Y. Chen G. (2017). Ferroptosis, a new form of cell death, and its relationships with tumourous diseases. J. Cell. Mol. Med. 21, 648–657. doi: 10.1111/jcmm.13008, PMID: 27860262 PMC5345622

[ref48] Zarjou A. Bolisetty S. Joseph R. Traylor A. Apostolov E. O. Arosio P.. (2013). Proximal tubule H-ferritin mediates iron trafficking in acute kidney injury. J. Clin. Invest. 123, 4423–4434. doi: 10.1172/JCI67867, PMID: 24018561 PMC3784534

[ref49] Zhang W. Gai C. Ding D. Wang F. Li W. (2018a). Targeted p53 on small-molecules-induced Ferroptosis in cancers. Front. Oncol. 8:507. doi: 10.3389/fonc.2018.00507, PMID: 30450337 PMC6224449

[ref50] Zhang R. Ji J. Zhou X. Li R. (2020). Irisin Pretreatment protects kidneys against acute kidney injury induced by ischemia/reperfusion via Upregulating the expression of uncoupling protein 2. Biomed. Res. Int. 2020:6537371. doi: 10.1155/2020/3170927, PMID: 32934963 PMC7479469

[ref51] Zhang D. Liu Y. Wei Q. Huo Y. Liu K. Liu F.. (2014). Tubular p53 regulates multiple genes to mediate AKI. J. Am. Soc. Nephrol. 25, 2278–2289. doi: 10.1681/ASN.2013080902, PMID: 24700871 PMC4178437

[ref52] Zhang J. McCullough P. A. (2016). Lipoic acid in the prevention of acute kidney injury. Nephron 134, 133–140. doi: 10.1159/000448666, PMID: 27603173

[ref53] Zhang Y. H. Wang D. W. Xu S. F. Zhang S. Fan Y. G. Yang Y. Y.. (2018b). Alpha-Lipoic acid improves abnormal behavior by mitigation of oxidative stress, inflammation, ferroptosis, and tauopathy in P301S tau transgenic mice. Redox Biol. 14, 535–548. doi: 10.1016/j.redox.2017.11.001, PMID: 29126071 PMC5684493

[ref54] Zhao W. Zhang L. Chen R. Lu H. Sui M. Zhu Y.. (2018). SIRT3 protects Against acute kidney injury via AMPK/mTOR-regulated autophagy. Front. Physiol. 9:1526. doi: 10.3389/fphys.2018.01526, PMID: 30487750 PMC6246697

