# Supplementary figures and images for: A-Lipoic Acid Alleviates Folic Acid-Induced Renal Damage Through Inhibition of Ferroptosis
Source: Front Physiol. 2021 Sep 17;12:680544. doi: 10.3389/fphys.2021.680544 (PMC8493959; doi:10.3389/fphys.2021.680544)

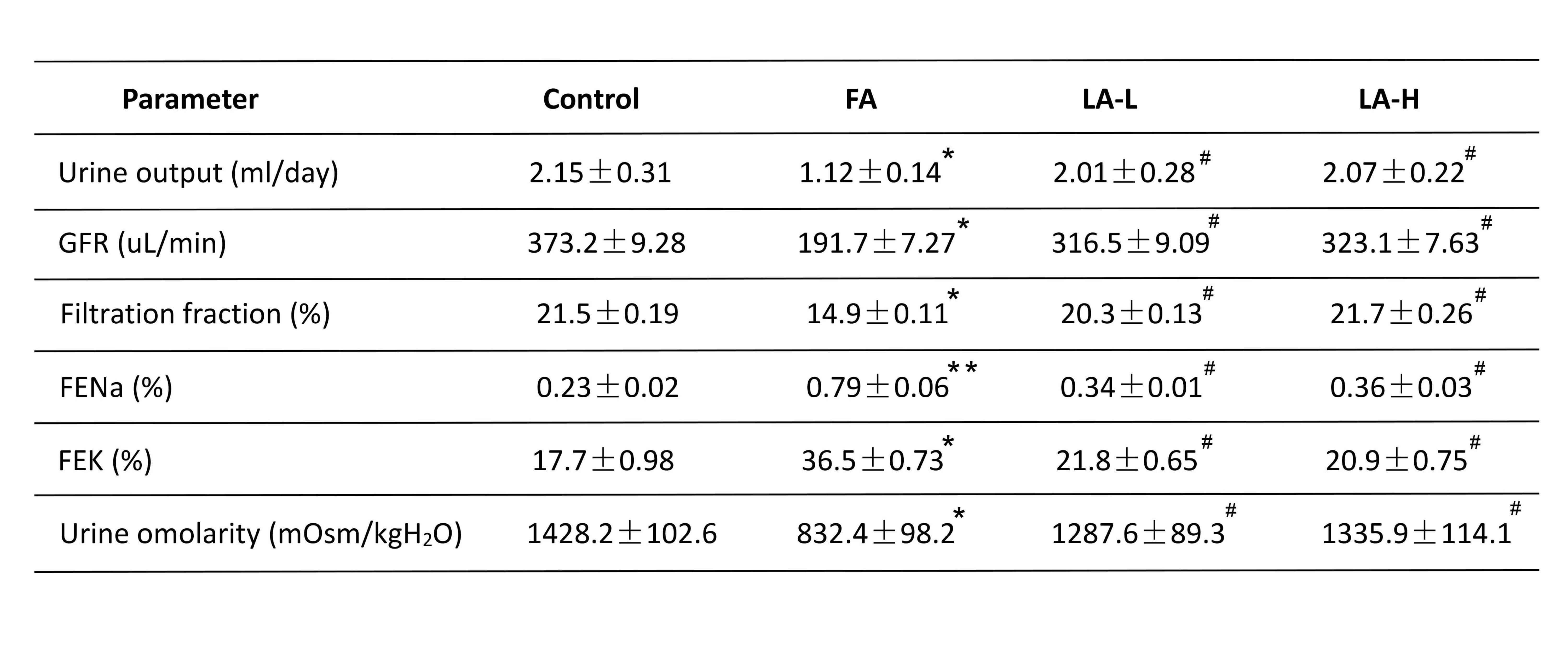

Supplement: Supplementary Table 1 — Changes in renal function parameters due to FA overdose injection in mice and effect of LA supplementation. Data are collected after housing the mice in metabolic cages for the last 24h. Urinary volume are obtained in individual metabolic cage. GFR mean glomerular filtration rate; Flitration fraction=GFR/plasma flow; and FENa mean fractional excretion of sodium and FEK mean fractional excretion of potassium. Data are presented as mean±SE (N=5). For the FA group vs. the control group, *indicates p<0.05, and **indicates p<0.01. For the LA-treated groups vs. the FA group, #indicates p<0.05, and ##indicates p<0.01. [file Image_1.jpg]

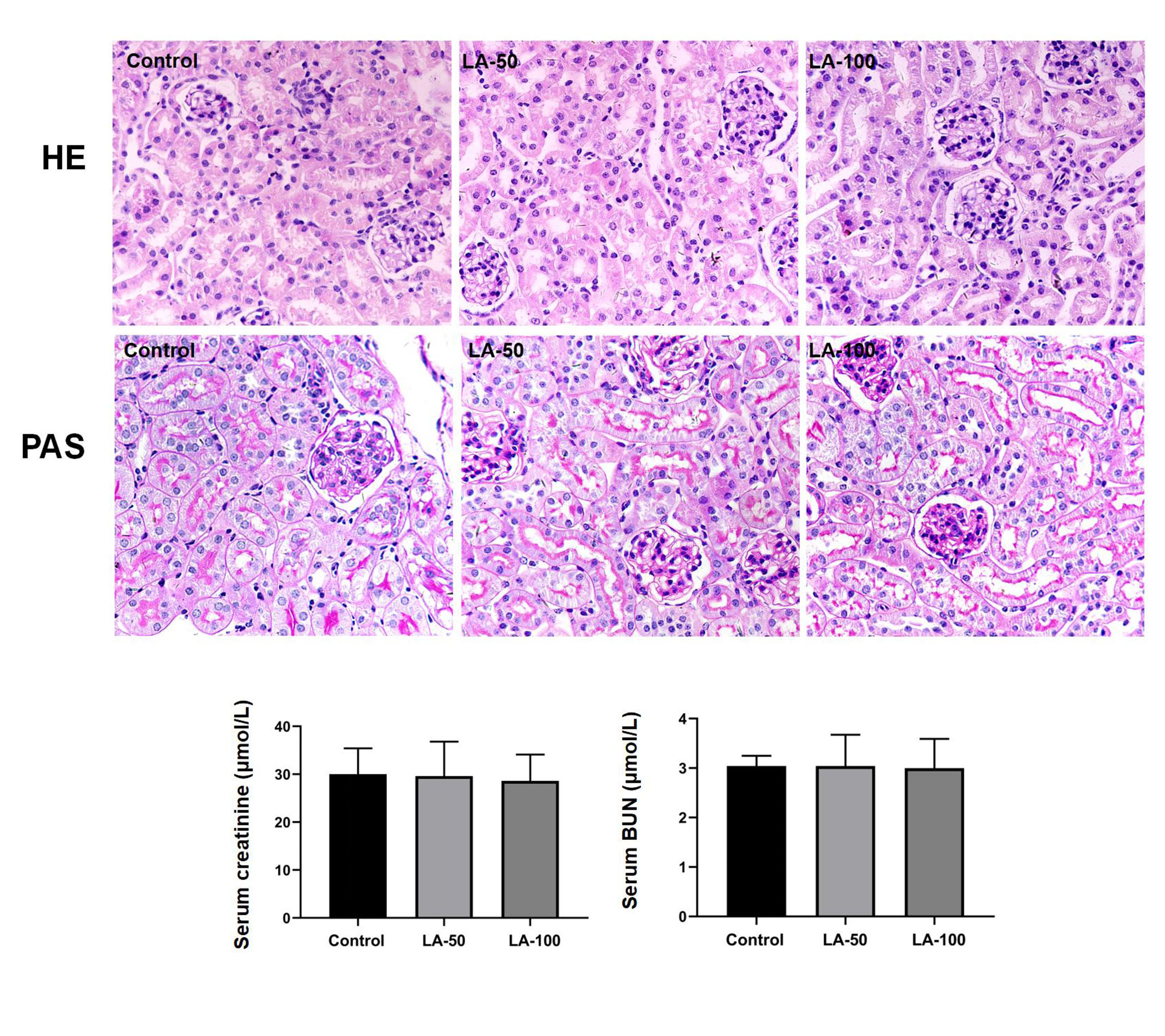

Supplement: Supplementary Figure 1 — No changes of renal morphology and function in the healthy mice after oral medications of LA (50 or 100mg/kg). The healthy mice are given an oral administration of LA at 50mg/kg (LA-50) or 100mg/kg (LA-100). (A) Representative images of H&E staining, showing histological changes of healthy mice without or with LA 50mg/kg (LA-50) or LA 100mg/kg (LA-100) oral administration. (B) Representative images of PAS staining, showing histological changes of healthy mice without or with LA 50mg/kg (LA-50) or LA 100mg/kg (LA-100) oral administration. The renal function is evaluated by (C) serum creatinine and (D) serum BUN levels. [file Image_2.jpg]

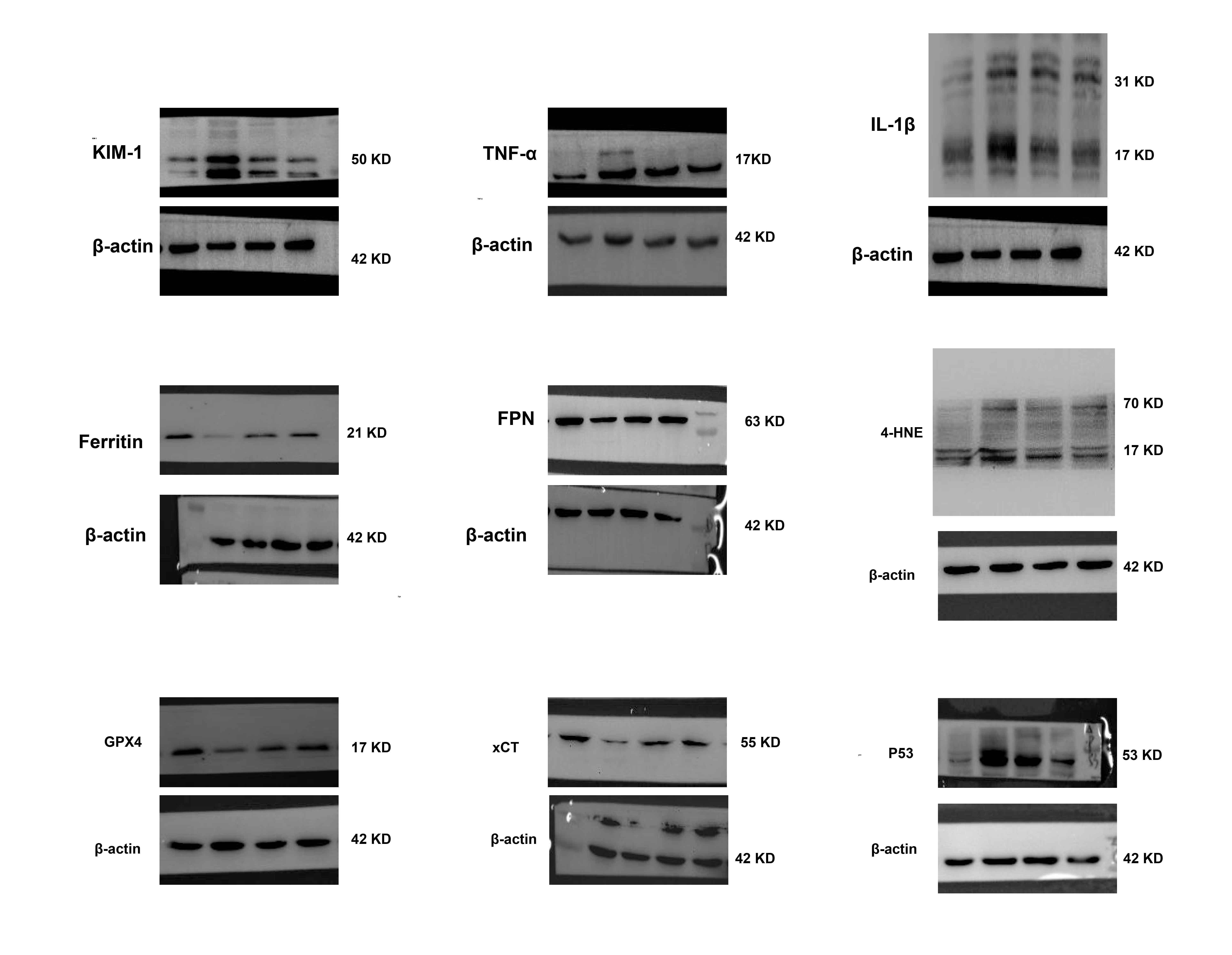

Supplement: Supplementary Figure 2 — The original data of the western blotting. [file Image_3.jpg]
